# Evaluation of the cause of unexplained radiocaesium contamination of brown rice in Fukushima in 2013 using autoradiography and gamma-ray spectrometry

**DOI:** 10.1038/srep20386

**Published:** 2016-02-04

**Authors:** Hisaya Matsunami, Toshifumi Murakami, Hideshi Fujiwara, Takuro Shinano

**Affiliations:** 1 Agricultural Radiation Research Center, NARO Tohoku Agricultural Research Center, Fukushima 9602156, Japan; 2 Technical Support Section 4, NARO Tohoku Agricultural Research Center, Fukushima 9602156, Japan; 3 Soil Environment Division, National Institute for Agro-Environmental Sciences, Tsukuba 3058604, Japan

## Abstract

The Great East Japan Earthquake on 11 March 2011, caused the release of radioactive materials from the Fukushima Daiichi Nuclear Power Plant (FDNPP), contaminating eastern Japan, particularly in part of Fukushima Prefecture. In 2012 and 2014, the radiocaesium concentration in brown rice did not exceed regulatory levels in Minamisoma City, Fukushima. However, in 2013, some radiocaesium concentrations in brown rice exceeded regulatory levels. In this work, autoradiograms showed that high radioactivity was present as contaminated spots on the panicles of rice and in brown rice in 2013. We evaluate the contribution of direct contamination to the radiocaesium concentration in brown rice and discuss the origin of radiocaesium contamination in brown rice using the ^134^Cs/^137^Cs radioactivity ratio. Here, we show that the main cause of the unexplained radiocaesium contamination of brown rice in Minamisoma City in 2013 is the adherence of radioactive materials to the rice panicles, and these radioactive materials are associated with reactor units 2 or 3 of FDNPP.

The Fukushima Daiichi nuclear disaster caused by the Great East Japan Earthquake on 11 March 2011, released a large amount of radionuclides that polluted a wide area of eastern Japan. The contamination is of great concern for Japanese food safety. Immediately after the accident, the major radionuclides found in plants and environmental resources, such as soil and water, were ^134^Cs (half-life: 2.07 years), ^137^Cs (half-life: 30.1 years), and ^131^I (half-life: 8.02 days). By now, all the radioiodine has decayed. Thus, radiocaesium, particularly ^137^Cs, is of the greatest concern due to its relatively long half-life.

The Japanese Ministry of Health, Labor and Welfare (MHLW) has set provisional regulatory levels of 500 Bq kg^−1^ for radionuclides in food, and the updated level of radiocaesium was set at 100 Bq kg^−1^ for general food, excluding infant food, milk, water, and beverages, in April 2012^1^. In Fukushima Prefecture, radiocaesium contamination in agricultural products is closely monitored before the products are shipped. The inspection of radiocaesium contamination in the rice produced in Fukushima Prefecture after the nuclear disaster in 2011 has been summarized by Nihei *et al*.^2^ Briefly, in Fukushima Prefecture, all brown rice in all bags for shipment must be inspected using a belt-conveyor-type radiocaesium concentration tester equipped with NaI or other types of scintillation counters^2^,^3^. In 2012 and 2013, 0.0007% and 0.0003% of rice bags exhibited values over 100 Bq kg^−1^, respectively^4^. However, none of the rice bags in 2014 had levels over 100 Bq kg^−1^ due to the implementation of effective countermeasures for inhibiting further uptake of radiocaesium by plants, such as the use of potassium fertilizer^3^.

In 2013 in Fukushima Prefecture, 28 of the approximately 11 million sampled bags of rice were identified as having excessive amounts of radiocaesium. Despite the use of countermeasures, 27 of the 28 bags had been produced in a limited area of Minamisoma City^4^. In this area, the radiocaesium concentration in brown rice did not exceed 100 Bq kg^−1^ in 2012 and 2014^4^,^5^. The attachment of radioactive materials to the leaves and panicles of rice was detected by means of autoradiography, suggesting that the causes of the radiocaesium contamination of brown rice may be direct contamination (adherence to the aerial parts of plants and plant-based absorption) in addition to indirect contamination (absorption from soil and irrigation water)^6^.

In this report, we evaluate the effect of the direct contamination on the radiocaesium concentration in brown rice in Minamisoma City during 2013 and discuss the origin of the radiocaesium contamination of brown rice by examining the ^134^Cs/^137^Cs radioactivity ratio.

## Results and Discussion

### Rice radiocaesium contamination in 2013

**Figure 1:**
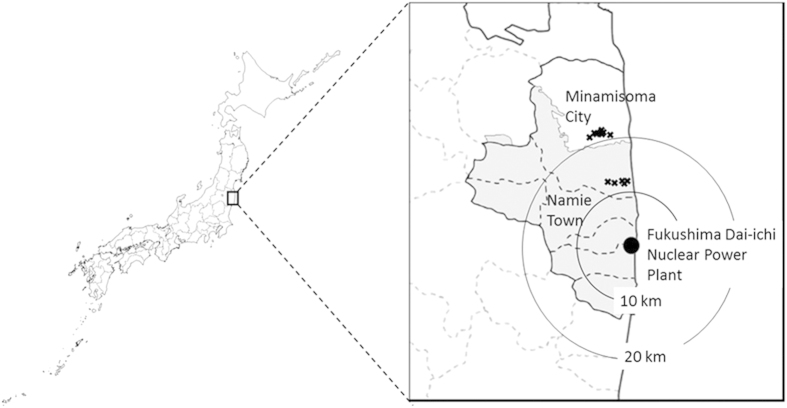
Map showing the radiocaesium contamination in brown rice in Minamisoma City in 2013. The grey areas represent areas subject to the evacuation order. The × symbols mark places where radioactivity concentrations in brown rice were over 100 Bq kg^−1^. The map was generated based on the data from the Ministry of Agriculture, Forestry and Fisheries of Japan (MAFF)^4^. The map was created using Adobe Photoshop Elements 13 and Microsoft PowerPoint 2013 software.

The area located up to 20 km north of the Fukushima Daiichi Nuclear Power Plant (FDNPP), including the southern part of Minamisoma City and neighbouring Namie town, was subjected to an evacuation order (1 Oct 2014), and only rice farming for scientific studies in the selected paddy fields was permitted (Fig. 1)^4^. Thus, the central area of Minamisoma City coincided with the southern limit of the area north of FDNPP available for farming rice for human consumption.

Brown rice with radiocaesium concentrations exceeding 100 Bq kg^−1^ in 2013 was detected in the southern area and part of the central area of Minamisoma City, located 12–21 km away from the FDNPP (Fig. 1)^4^. In the central area, where radiocaesium levels exceeding 100 Bq kg^−1^ were detected in 27 rice bags before shipment, the radiocaesium concentrations were higher in 2013 and lower in 2014^4^,^5^. Of 1,589 bags, 43% had radiocaesium concentrations exceeding 50 Bq kg^−1^ in 2013^4^, whereas none of the 1,955 bags had concentrations exceeding 50 Bq kg^−1^ and only one bag had a concentration exceeding 25 Bq kg^−1^ in 2014^4^. The transfer factors (TFs) of radiocaesium from paddy field soil to brown rice (TF = radiocaesium concentration in brown rice (Bq kg^−1^)/radiocaesium concentration in the paddy field soil (Bq kg^−1^)) in the southern area are shown in Table 1. The TFs in 2013 ranged from 0.06 to 0.58 (Table 1). According to Kato *et al*.^7^ the TF values of brown rice ranged from 0.00082 to 0.037 in Fukushima and neighbouring prefectures in 2011 shortly after the FDNPP accident. The TF values in 2014 were within the range of values reported by Kato *et al*.^7^, whereas those in 2013 were much higher (Table 1). Therefore, it is not plausible that indirect contamination was the main source of the contamination of brown rice in Minamisoma City in 2013.

### Cause of rice radiocaesium contamination

**Figure 2:**
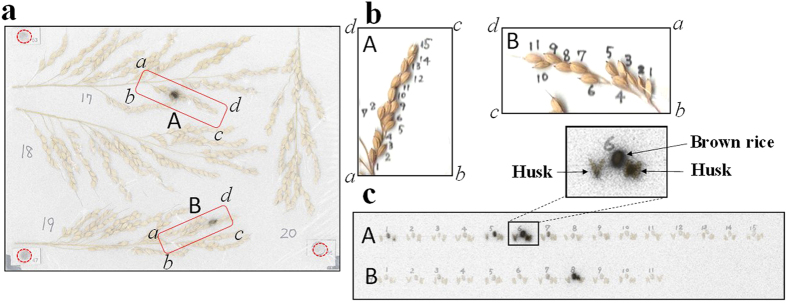
Autoradiography of rice in 2013. (**a**) Superimposition of autoradiogram and photograph of panicles. The dotted red circles are the potassium chloride markers. (**b**) Photographs of the rachis branches surrounded by red dotted rectangles A and B in Fig. 2a. (**c**) Superposition of autoradiogram and photograph of brown rice and husks. The sample numbers of the unhulled rice are shown and correspond to those in Fig. 2b.

Figure 2 shows autoradiograms of the panicles, brown rice, and husks of the highly contaminated rice samples collected in 2013. Black dots on the autoradiograms indicate the presence of radioactive materials. High-intensity spots were visible on the panicles (Fig. 2a). Brown rice also showed high radioactivity at positions corresponding to the high-intensity spots on the panicles (Fig. 2c). In all 40 brown rice samples with high levels of radiocaesium contamination in the southern area and part of the central area in 2013, high radioactivity was observed as numerous spots on the autoradiogram. In contrast to the samples in 2013, no radioactive spots were observed on the brown rice samples in 2014.

To evaluate the effect of the direct contamination on the radiocaesium concentration in brown rice in Minamisoma City in 2013, the grains of the brown rice samples with high radiocaesium concentrations in the southern area were divided into highly contaminated grains and less contaminated grains after 2 weeks of exposure on an imaging plate. Brown rice grains at positions corresponding to visible spots on the autoradiogram were isolated as highly contaminated grains. The radiocaesium concentrations in the highly contaminated grains were 17- to 48-fold (average of 29-fold) higher than those in the less contaminated grains (Table 1). If the radiocaesium in the highly contaminated grains originated from soil, irrigation water, or foliar contamination, the distribution of radiocaesium concentrations on the panicles and the brown rice should be even higher (Fig. 2a,c and Table 1). The radiocaesium contamination can be attributed to the contamination of the flowers because the radioactive materials that adhere to the rice flowers are directly incorporated into rice grains, which may cause a substantial increase in the radiocaesium concentration of rice grains^8^.

Although the highly contaminated grains contributed to 2–8% of the total weight, the percentage contribution of the highly contaminated grains to the total radiocaesium activity was estimated to be more than 41%, and the removal of highly contaminated grains caused a large decrease in the radiocaesium concentration in the brown rice samples (Table 1). These results show that the adherence of radioactive materials to the rice panicles was the main cause of the radiocaesium contamination.

### Origin of radiocaesium on the panicles

**Figure 3:**
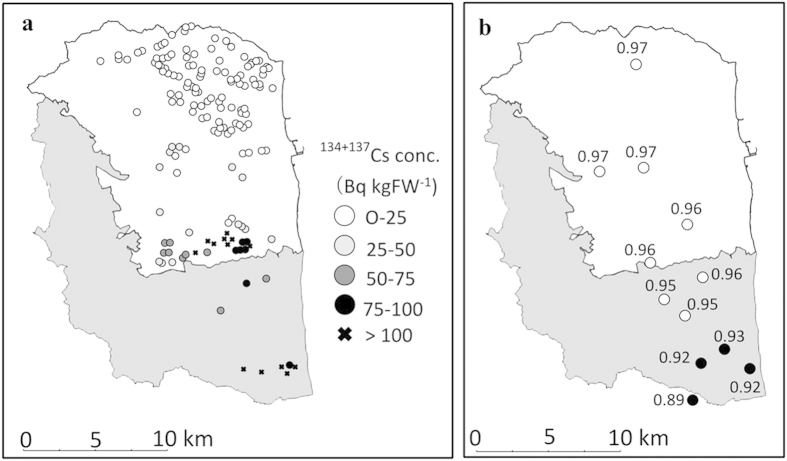
Spatial distribution of ^134 + 137^Cs concentrations in brown rice in 2013 and ^134^Cs/^137^Cs radioactivity ratio of paddy field soil. Grey areas represent areas subject to the evacuation order. (**a**) ^134+137C^s concentrations (Bq kg^-1^) of brown rice in 2013 and (**b**) ^134^Cs/^137^Cs radioactivity ratios of paddy field soil. Figure 3a was generated based on the data from the Ministry of Agriculture, Forestry and Fisheries of Japan (MAFF)^3^. The map was created using Adobe Photoshop Elements 13 and Microsoft PowerPoint 2013 software.

If the radioactive materials that adhered to the rice in 2013 originated from the areas where the radiocaesium concentration levels exceeded 100 Bq kg^−1^, the radiocaesium contamination in brown rice should also have been present in 2014 and in subsequent years. However, highly contaminated grains were not found in 2014. Therefore, the radioactive materials are not likely to have originated from these areas. Figure 3a shows the spatial distribution of the radiocaesium concentrations in brown rice in 2013 reported by the Ministry of Agriculture, Forestry and Fisheries of Japan (MAFF)^4^. According to this report, the radiocaesium concentrations in brown rice were highest in the southern area of Minamisoma City, and they generally decreased towards the northern area, suggesting that the source of radiocaesium contamination in 2013 was the southern area of Minamisoma City.

In the Fukushima Daiichi nuclear disaster on March 2011, hydrogen explosions occurred in reactor units 1, 2, and 3, releasing radioactive materials into the environment. Komori *et al*.^9^ reported that the ^134^Cs/^137^Cs radioactivity ratios of reactor units 1, 2, and 3 were 0.91 (0.89–0.93), 1.00 (0.96–1.05), and 1.01 (0.97–1.04), respectively. Therefore, these values could be used to identify the origin of the radiocaesium contamination. Figure 3b shows the spatial distribution of the ^134^Cs/^137^Cs radioactivity ratio of paddy field soil collected over a wide area of Minamisoma City. The ^134^Cs/^137^Cs radioactive ratio was time-corrected for each radioactive half-life to the value of the FDNPP accident on March 11, 2011, as described by Komori *et al*.^9^ The values of the ^134^Cs/^137^Cs radioactivity ratio of the paddy field soil ranged from 0.89 to 0.97 and gradually increased from the southern area to the northern area (Fig. 3b). These results indicate that the direct deposition of the radioactive aerosol released from the reactor unit of FDNPP on March 2011 varied across Minamisoma City. Our results showed that the paddy field soils in the southernmost part of the city were strongly affected by radioactive materials released from reactor unit 1.

If the radiocaesium in the brown rice was from the paddy field soil, the values of the ^134^Cs/^137^Cs radioactivity ratio of brown rice should be close to those of the paddy field soils because no isotope effect for caesium exists in the natural environment. The ^134^Cs/^137^Cs radioactivity ratios of brown rice in the southernmost section of the city in 2014 were close to those of the paddy field soil and similar to the ratio for reactor unit 1 (Table 3). In contrast, in 2013, the average value of the ^134^Cs/^137^Cs radioactivity ratio of highly contaminated grains was approximately 1.0, which was significantly higher than that of paddy field soil (0.92) and similar to the ratios for reactor units 2 and 3 (Table 3). These results show that in the southernmost section of Minamisoma City, the radioactive materials that adhered to the panicles in 2013 were not from the paddy field soil but rather were associated with reactor units 2 and 3 of FDNPP. Although it is difficult to estimate the origin of radiocaesium contamination using the ^134^Cs/^137^Cs radioactivity ratio, in areas where the radioactive materials associated with reactor units 2 and 3 contaminated the grains and the paddy field soils (Fig. 3b), the ^134^Cs/^137^Cs radioactivity ratio of the highly contaminated grains in 2013 was also approximately 1.0.

Based on these results, we conclude that the main cause of the unexplained radiocaesium contamination of brown rice in Minamisoma City in 2013 was the adherence of radioactive materials to the rice panicles, and these radioactive materials, which appear to have originated from the southern area of Minamisoma City when the rice was flowering, were associated with reactor units 2 or 3 of FDNPP.

On 19 August 2013, the air dose rates increased at five measuring points 2.8 to 8.3 km north-northwest on the leeward side of FDNPP^10^, and high activity concentrations in the air were detected at the air filter station in the central area of Minamisoma City in the week of 15 August to 22 August 2013^11^. In the same week, debris removal operations were conducted at reactor unit 3 of FDNPP, and radioactive dust under the debris was spread outside of FDNPP^12^. Although the origin of radiocaesium on the panicles in 2013 is still unclear, it is likely that the debris removal operations led to the unexplained radiocaesium contamination of brown rice.

## Methods

We identified radiocaesium contamination in rice by autoradiography and estimated the ^134^Cs/^137^Cs radioactivity ratio in brown rice and soil samples by gamma-ray spectrometry.

### Autoradiography

The panicle and brown rice samples were collected from areas of Minamisoma City where radiocaesium concentrations exceeding 100 Bq kg^−1^ were detected in 2013. An imaging plate (BAS-SR2040, Fuji-Film, Japan) and a reading system (Typhoon FLA 7000, GE Healthcare Bio-Science Corp., USA) were used to obtain and analyse the images of panicles and brown rice samples. The panicle samples were fixed onto 42 × 30-cm cardboard pieces with cellophane tape. After covering the samples with polyvinylidene chloride resin film (Saran wrap, AsahiKASEI, Japan), potassium chloride markers were attached to the corners of the film to allow the autoradiogram and the photograph (or the cardboard-mounted samples) to be superimposed. The markers were made by packing potassium chloride (10 or 26 mg) into a frame, which was created by cutting a silicon tube with a 6-mm inside diameter into a 1-mm-thick round slice. The filled frame was sealed inside a laminated pouch. The cardboard-mounted panicle samples were exposed to an imaging plate in the dark for 2 days to evaluate the contamination state, and the imaging plates were scanned at a spatial resolution of 25 μm with the reading system. The unhulled rice on the rachis branch was separated into brown rice and husks. The brown rice and husks were fixed onto cardboard with double-sided tape and exposed to an imaging plate for 3 days.

To evaluate the effect of the direct contamination of brown rice by radiocaesium, the brown rice grains in each sample from the southern area of Minamisoma City with high levels of radiocaesium contamination were divided into highly contaminated grains and less contaminated grains. Brown rice (80 g, approximately 4000 grains) was affixed to a piece of cardboard with spray adhesive (3 M Super 77 Multipurpose Spray Adhesive, 3 M Japan Limited, Japan) and then exposed to an imaging plate for 2 weeks to isolate the highly contaminated grains as much as possible. Visible spots with the radioactivity in the samples and the markers on the autoradiogram of brown rice were traced onto the clear plastic sheet, and the sheets were superimposed accurately on the cardboard-mounted brown rice samples at marked positions. The spots on the autoradiogram were several times larger than the actual grains due to the sample thickness and because the radiation was emitted in all directions. Therefore, it was difficult to distinguish the highly contaminated grains from neighbouring less contaminated grains. Accordingly, the grains of interest were selected, set at appropriate intervals on another piece of cardboard with double-sided tape, and exposed to an imaging plate for a further 2 weeks to confirm their high radioactivity. In total, 320–400 g of each of the brown rice samples was used to identify highly contaminated grains. Each group was weighed, pulverized, and then placed in a cylindrical polypropylene container (U-8 container, 65 mm in height and 50 mm in diameter).

### Gamma-ray spectrometry

The radionuclide concentrations and the ^134^Cs/^137^Cs radioactivity ratio of the brown rice and soil samples were determined with high-purity germanium (HPGe) detectors with relative efficiencies of 25% and 40%, respectively (GC2520-7500SL and GC4020-7500SL, Canberra, USA). The radiocaesium concentrations and ^134^Cs/^137^Cs radioactivity ratio of the brown rice samples in the study area were measured with a U-8 container and a 0.7 L Marinelli beaker, respectively, in 2013 and 2014.

Paddy field soil samples from the surface to a depth of 15 cm were collected over a wide area of Minamisoma City. Soil samples were air-dried and passed through a 2-mm screen, and plant residues were removed prior to gamma-ray spectroscopy. The radiocaesium concentrations and ^134^Cs/^137^Cs radioactivity ratio of the soil samples were measured in a U-8 container.

The concentration of ^137^Cs was directly determined using gamma lines at 661.6 keV. In contrast to ^137^Cs, ^134^Cs emits multiple gamma-rays. The largest emission rate of ^134^Cs gamma-rays was 98% at 604.7 keV and 86% at 795.9 keV. The ^134^Cs concentration is determined from the weighted average of gamma-ray counting at the two gamma lines^13^. By means of gamma-ray counting for a long period of time, the counting uncertainties (relative standard deviation) of ^134^Cs and ^137^Cs were maintained below 1%, except for the less contaminated grains in 2013. Counting times were from 81,794 to 1,843,654 s for brown rice samples (the highly contaminated grains in 2013 and brown rice in 2014) and from 3,788 to 309,033 s for soil samples. The detection efficiencies of the HPGe detectors were determined with approximately 5% uncertainty using a set of activity standard volume sources (MX-033, Japan Isotope Association). This uncertainty uniformly contributes to the systematic error of all measurement results and can be distinguished from the random error due to the counting of gamma-rays emitted from a sample. The self-absorption of gamma-rays was adequately corrected based on the thickness and density of the sample and the attenuation coefficients of gamma-rays. Furthermore, the coincidence-summing correction of gamma-rays emitted from ^134^Cs was performed using the peak-to-total calibration method^14^. The radionuclide concentrations in the samples were time-corrected to the values on 1 October of each year, the conventional harvest time for rice in Fukushima, by each radioactive half-life. However, the ^134^Cs/^137^Cs radioactivity ratio of the samples was corrected to the values on 11 March 2011, the time on which the nuclear reactor at the FDNPP was scrammed. The uncertainty of the ^134^Cs/^137^Cs ratio was calculated by means of the propagation of counting uncertainties.

## Additional Information

**How to cite this article**: Matsunami, H. *et al*. Evaluation of the cause of unexplained radiocaesium contamination of brown rice in Fukushima in 2013 using autoradiography and gamma-ray spectrometry. *Sci. Rep.* **6**, 20386; doi: 10.1038/srep20386 (2016).

## Figures and Tables

**Table 1:** Radiocaesium concentrations in brown rice and paddy field soil in the southern area of Minamisoma City.

Year	Sample ID	^134 + 137^Cs conc. (Bq kg^-1^)*1				Transfer factor
		Brown rice			Paddy field soil	
		HG*2	LG*2	Average		
2013	MSG1	1,628.2 ± 7.8	59.5 ± 0.3	152	315.3 ± 1.9	0.4824
	MSG4	2,220.4 ± 11.4	49.3 ± 0.2	167	290.4 ± 1.8	0.5767
	MSG7	1,271.3 ± 6.9	68.4 ± 0.4	161	427.8 ± 2.0	0.3754
	MSG8	1,380.4 ± 6.0	79.7 ± 0.5	172	1,792.1 ± 8.3	0.0960
	MSG11	1,345.6 ± 6.0	36.7 ± 0.2	80	449.9 ± 2.1	0.1788
	MSG14	843.2 ± 3.9	40.0 ± 0.4	76	510.9 ± 2.4	0.1481
	MSG17	1,197.2 ± 6.2	55.5 ± 0.3	106	1,054.8 ± 5.7	0.1001
	MSG18	1,590.5 ± 2.1	33.2 ± 0.7	72	1,222.5 ± 5.8	0.0590
	MSG19	938.3 ± 3.2	29.9 ± 0.6	50	619.8 ± 2.9	0.0805
	MSG20	1,319.1 ± 2.0	47.6 ± 1.0	115	369.4 ± 1.7	0.3111
2014	MSG38	−	−	18.5 ± 0.1	1,590.7 ± 7.2	0.0116
	MSG39	−	−	3.7 ± 0.0	2,144.9 ± 9.5	0.0017
	MSG41	−	−	24.2 ± 0.1	1,860.7 ± 8.4	0.0130

^*1^Measured value ± counting error. The radionuclide concentrations were corrected to the values on 1 October of each year.

^*2^HG: highly contaminated grains. LG: less contaminated grains.

**Table 2:** Percentage contribution of the highly contaminated grains to the total weight and the total radiocaesium activity in the southern area of Minamisoma City in 2013.

Sample ID	Percentage contribution			
	To the total weight (%)		To the total activity (%)	
	HG *	LG*	HG *	LG*
MSG1	5.9	94.1	63.2	36.8
MSG4	5.4	94.6	72.2	27.8
MSG7	7.7	92.3	60.6	39.4
MSG8	7.1	92.9	57.0	43.0
MSG11	3.3	96.7	55.9	44.1
MSG14	4.4	95.6	49.4	50.6
MSG17	4.4	95.6	49.8	50.2
MSG18	2.5	97.5	55.1	44.9
MSG19	2.2	97.8	41.4	58.6
MSG20	5.3	94.7	60.8	39.2

^*^HG: highly contaminated grains. LG: less contaminated grains.

**Table 3:** ^134^Cs/^137^Cs radioactivity ratio in brown rice and paddy field soil in the southernmost section of Minamisoma City*1.

Year	Sample ID	Brown rice		Paddy field soil
		HG*2	Average	
2013	MSG1	0.996 ± 0.010	—	0.912 ± 0.011
	MSG4	0.990 ± 0.011	—	0.923 ± 0.012
	MSG7	0.999 ± 0.011	—	0.914 ± 0.009
	MSG8	1.017 ± 0.009	—	0.908 ± 0.009
	MSG11	1.004 ± 0.009	—	0.940 ± 0.009
	MSG14	1.007 ± 0.010	—	0.926 ± 0.009
	MSG17	1.004 ± 0.011	—	0.921 ± 0.010
2014	MSG38	—	0.938 ± 0.009	0.919 ± 0.009
	MSG39	—	0.910 ± 0.011	0.885 ± 0.008
	MSG41	—	0.937 ± 0.009	0.936 ± 0.009

^*1^Ratio value ± uncertainties calculated by means of propagation of counting errors. The ^134^Cs/^137^Cs radioactivity ratios of the samples were corrected to the values on 11 March 2011.

^*2^HG: highly contaminated grains.
